# Molecular Characterization of Vitellogenin and Its Receptor in *Sogatella furcifera*, and Their Function in Oocyte Maturation

**DOI:** 10.3389/fphys.2019.01532

**Published:** 2019-12-19

**Authors:** Kui Hu, Ping Tian, Yan Tang, Lu Yang, Lin Qiu, Hualiang He, Wenbing Ding, Zhongcai Li, Youzhi Li

**Affiliations:** ^1^Hunan Provincial Key Laboratory for Biology and Control of Plant Diseases and Insect Pests, College of Plant Protection, Hunan Agricultural University, Changsha, China; ^2^National Research Center of Engineering & Technology for Utilization of Botanical Functional Ingredients, Hunan Agricultural University, Changsha, China; ^3^Hunan Provincial Engineering & Technology Research Center for Biopesticide and Formulation Processing, Hunan Agricultural University, Changsha, China; ^4^Plant Protection and Inspection Station, Agricultural Bureau of Hanshou County, Hanshou, China

**Keywords:** *Sogatella furcifera*, vitellogenin, vitellogenin receptor, reproduction, RNA interference

## Abstract

The yolk protein precursor, vitellogenin (Vg), provides nutrition for embryonic development whereas the vitellogenin receptor (VgR) is responsible for the uptake of yolk protein by maturing oocytes. These two proteins are key reproduction-related proteins in insects. We cloned and characterized *Vg* and *VgR* genes in *Sogatella furcifera*, and investigated their function in oocyte maturation. Cloned *SfVg* and *SfVgR* have open reading frames of 6,114 and 5,796 bp, encoding 2,037 and 1,931 amino acid residues, respectively. Structural analysis indicates that SfVg has the three conserved LPD_N, DUF1943, and VWFD domains, SfVgR contains all conservative motifs of the LDLR superfamily. Both genes were highly expressed in adult females; *SfVg* was most highly expressed in the fat body whereas *SfVgR* was mainly expressed in the ovary. Knockdown of either gene reduced yolk protein deposition in oocytes and arrested oocyte maturation. However, silencing one of these two genes did not affect the transcript level of the other. These results demonstrate the role of SfVgR in transporting SfVg into oocytes. Both SfVg and SfVgR are essential for oocyte maturation in *S. furcifera* and both genes could potentially be targeted as means of controlling this pest.

## Introduction

Ovarian development is essential for reproduction in oviparous insects (Cong et al., 2015). Embryonic development requires large amounts of yolk protein. Vitellogenin (Vg), a yolk protein precursor, is mainly synthesized in the fat body and released into the hemolymph after being modified by glycosylation, phosphorylation, proteolytic cleavage, sulphated into glycolipoproteins and cleaved into large and small subunits (Tufail and Takeda, 2008). Vg is taken up into developing oocytes by vitellogenin receptor (VgR) mediated endocytosis (Giorgi et al., 1999). Vg is then transported to recipient cells by VgR where it provides nutrients essential for embryonic development (Rodenburg et al., 2006).

In insects, there are one to three *Vg* genes amongst different species (Lee et al., 2000; Tufail et al., 2010; Terrapon et al., 2014). Vgs are large protein molecules of ∼200 kDa (Lee et al., 2015) that belong to the large lipid transfer protein (LLTP) superfamily. They generally have a ∼20 residue putative signal peptide, a lipid binding domain (LPD_N) in the N-terminal region, a domain of unknown function (DUF1943), and a von Willebrand factor type D domain (VWD) in the C-terminal region (Tufail and Takeda, 2008; Ibanez et al., 2017; Guo et al., 2018; Shang et al., 2018). Most insects have only a single *VgR* gene encoding a polypeptide with a molecular weight of 180–214 kDa (Sappington and Raikhel, 1998). VgR belongs to the low-density lipoprotein receptor (LDLR) family (Tufail and Takeda, 2009; Zhao et al., 2018). The amino acid sequences of VgR include five highly conserved and functionally different amino acids domains; a ligand binding domain (LBD), an epidermal growth factor-precursor domain (EGF), an O-linked sugar domain, low-density lipoprotein-receptor Tyr-Trp-Thr-Asp (YWTD) repeats and a transmembrane domain (Sappington and Raikhel, 1998; Tufail and Takeda, 2009; Shang et al., 2018; Yao et al., 2018).

Vitellogenin has been found to have several non-reproductive functions, such as differentiating worker and forage castes of eusocial insects and the regulation of hormonal dynamics, immunity and changes in gustatory responsiveness (Amdam et al., 2003; Guidugli et al., 2005; Nelson et al., 2007; Salmela et al., 2015; Wu et al., 2015). However, the primary role of Vg is to form yolk protein and provide the nutrients required for embryonic development. There is strong evidence that Vg and VgR are essential for successful reproduction in insects. For example, in *Nilaparvata lugens*, dsRNA-mediated silencing of *NlVg* and *NlVgR* arrested ovarian development causing infertility (Lu et al., 2015). Similarly, in *Aphis citricidus*, dsRNA knockdown of *AcVg* and *AcVgR* negatively affected embryonic and postembryonic development (Shang et al., 2018).

*Sogatella furcifera* is a highly fecund insect that causes major damage to rice crops in Asia. This hemipteran pest feeds on the phloem sap of rice plants resulting in delayed tillering, shriveled grain, stunted growth and plant death if infestations are sufficiently heavy (Zhou et al., 2013; Zhang G. et al., 2016). Furthermore, *S. furcifera* is a vector of the southern rice black-streaked dwarf virus (SRBSDV) (Zhou et al., 2008). SRBSDV causes rice plants to produce small spikes with few, or no, rice grains, thereby drastically reducing rice yields (Lv et al., 2017). It is strategic to control plant diseases by controlling disease vectors (Hu et al., 2019a). Since ovarian development is essential for the reproduction of insect pests, disrupting specific steps of oogenesis could be a way of controlling pest populations.

We first cloned *S. furcifera Vg (SfVg*) and *VgR* (*SfVgR*) sequences, analyzed their basic molecular and structural characteristics and compared them to those of other insects. In addition, we evaluated the expression patterns of *SfVg* and *SfVgR* in different tissues and developmental stages. Finally, we used dsRNA-mediated gene silencing to determine the role of Vg and VgR in oocyte maturation in *S. furcifera*.

## Materials and Methods

### Insect Collection and Rearing

*Sogatella furcifera* was collected from a rice field at Hunan Agricultural University, Changsha, China. More than three generations were reared on Fengyou No.9 rice seedlings in a climatic chamber at 26 ± 1°C, with a relative humidity of 80 ± 5% and under a 16:8 h (L:D) photoperiod.

Samples of the 1st (30 insects), 2nd (30 insects), 3rd (15 insects), 4th (10 insects), 5th instar (10 insects), and 96 h old female (5 insects) and male (5 insects) adults were randomly chosen to measure the expression of *SfVg* and *SfVgR* in the whole bodies of these different age classes. The expression of *SfVg* and *SfVgR* was also measured in the whole bodies of 0, 24, 48, 72, 96, and 132 h old female adults (five insects from each age group), and in the head, thorax, midgut, ovary and fat body of twenty 96 h old female adults. Each sample had three replicates.

### RNA Isolation and cDNA Synthesis

Total RNA was extracted using a MiniBEST Universal RNA Extraction Kit (TaKaRa, Dalian, China). A PrimeScript^TM^ RT reagent Kit with gDNA Eraser (TaKaRa) was used to synthesized first-strand cDNA with 0.5 μg total RNA in a 20 μl reaction mixture according to the manufacturer’s instructions.

### Sequence Comparisons and Phylogenetic Analysis

The sequences of *SfVg* and *SfVgR* were derived from transcriptome data and published genome sequences (Wang et al., 2017) and identified using the online BLAST program on the National Center for Biotechnology Information (NCBI) website^1^. The modular domains of SfVg and SfVgR were analyzed with the SMART program^2^ and the pI/Mw of the these two protein sequences predicted using the compute pI/Mw tool^3^. The NetNGlyc1.0 Server^4^ (NXS/T) was used to identify glycosylation sites. Phosphorylation sites were predicated using the NetPhos 3.1 Server^5^ and transmembrane (TM) regions using the TMHMM server v2.0^6^.

Amino acid sequences of Vgs from 32, and VgRs from 35 other insect species were downloaded from the GenBank database, the name and GenBank numbers of these 32 Vgs and 35 VgRs were listed in Supplementary File 1. ClustW was used to align amino acid sequences, after which a neighbor-joining phylogenetic tree with 1 000 bootstrap replicates was constructed using MEGA 5.0 (Tamura et al., 2011).

### Developmental Expression Profiles of SfVg and SfVgR

Quantitative RT-PCR (qRT-PCR) was used to detect the expression levels of *SfVg* and *SfVgR*. qRT-PCR was conducted on a CFX96 Touch^TM^ Real-Time PCR Detection System (Bio-Rad, Hercules, CA, United States) with TB Green Premix Ex Taq^TM^ II (TaKaRa) under the following conditions: 30 s denaturation at 95°C, followed by 40 × 5 s cycles at 95°C and finally 30 s at 60°C. The qRT-PCR primers of each gene were designed using the NCBI profile server2^7^. Primers and their amplification efficiencies are shown in Table 1. The relative expression levels of the target genes were calculated by normalizing their CQ values to those of α*-1 tubulin* (*SfTub*, accession No. KP735521) and *elongation factor 1*α (*SfEF1*α, accession No. KP735517) using the 2^–Δ^ ^Δ^ ^*Ct*^ method, like previous studies (Livak and Schmittgen, 2001; An et al., 2015; Yin et al., 2018; Yue et al., 2018). Each sample had three technological replicates.

**TABLE 1:** Primers used in this study.

**Purpose**	**Primer name**	**Primer sequence (5′→3′)**	**E (%)^a^**	***R*^2^**
qPCR	qVg-F	CACAAGGTTGCTTCTGGCATC	93.8	0.999
	qVg-R	TTGGCCAAAGCTAGAGTAGCC		
	qVgR-F	ACAAGAGCGATCCTGCCAAA	96.3	0.993
	qVgR-R	ATTCGATCCACTCGTGTCCG		
	qTub-F	GAGGACACTACACCATCGGC	93.6	0.995
	qTub-R	TCAACAGCGAGGTGAATCCG		
	qEF1α-F	AAGATCGGTTACAACCCGGC	103.8	0.989
	qEF1α-R	TCCTTGCGCTCAATGTTCCA		
RNAi	Vg-F	GGATCCTAATACGACTCACTATAGGAGGGCTTTGGAGATCTTGCC^b^	n.a.	n.a.
	Vg-R	GGATCCTAATACGACTCACTATAGGAGAGTTGGCTGGGTCCATTG		
	VgR-F	GGATCCTAATACGACTCACTATAGGACCACAGCCACCAACGATAG	n.a.	n.a.
	VgR-R	GGATCCTAATACGACTCACTATAGGACGTCAGGGGACGTAAACAC		
	EGFP-F	GGATCCTAATACGACTCACTATAGGGAGGACGACGGCAACTACAAG	n.a.	n.a.
	EGFP-R	GGATCCTAATACGACTCACTATAGGGGTCCATGCCGAGAGTGATCC		

*^*a*^PCR efficiency; ^*b*^T7 RNA polymerase promoter is underlined; n.a. = not applicable.*

### RNA Interference

RNA interference was used to investigate the function of SfVg and SfVgR in oocyte maturation with the *EGFP* gene (*enhanced green fluorescent protein*, GenBank Accession No. U55762) as a parallel control. For dsRNA preparation, *SfVg*, *SfVgR*, and *EGFP* genes were first amplified using specific primers (Table 1) conjugated with the T7 RNA polymerase promoter sequence. The resultant PCR products were used as templates to synthesize dsRNA (The length of dsVg, dsVgR and dsEGFP was 541, 526, and 441 bp, respectively) using the T7 RiboMAX Express RNAi System (Promega, Madison, WI, United States) according to the manufacturer’s protocol. Newly emerged female adult *S. furcifera* were anesthetized with CO_2_ for 90 s after which 100 ng (50 nL, 2000 ng/μL; according to the preliminary tests) of dsRNA was injected into each female with a Nanoinjector (Drummond Scientific, Pennsylvania, PA, United States) through the conjunctive between prothorax and mesothorax (Liu et al., 2010). Three replicate samples of five insects were randomly selected to assess the efficiency of RNAi treatment 48, 72, and 96 h after injection. The ovarian phenotypes of more than 20 females in each treatment group were observed and photographed using a stereomicroscope (Motic SMZ-161, Motic Group Co., Xiamen, China) equipped with a D3400 digital camera (Nikon, Tokyo, Japan) 132 h post-injection.

### Statistical Analysis

The statistical significance of differences in expression levels of *SfVg* and *SfVgR* in different developmental stages and tissues was assessed with one-way analysis of variance (ANOVA) followed by Tukey’s honestly significant difference test for multiple sample comparisons (*P* < 0.05). The statistical significance of differences in gene expression between the RNAi treatment and control groups was assessed using Student’s *t*-test (^∗^*P* < 0.05, ^∗∗^*P* < 0.01, ^∗∗∗^*P* < 0.001). Statistical analyses were performed using GraphPad Prism 8 software (GraphPad Software Inc., San Diego, CA, United States). All data are expressed as means ± standard errors (SE).

## Results

### Sequence and Structure of SfVg

**FIGURE 1:**
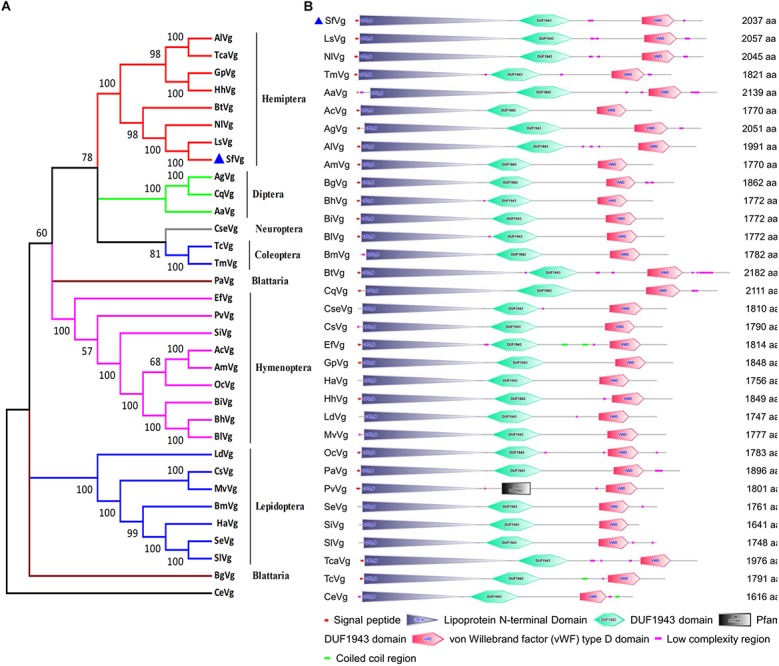
Phylogenetic tree and schematic of primary protein structures of *Sogatella furcifera* vitellogenin (Vg) and Vgs in other insects. **(A)** Phylogenetic tree constructed with MEGA 5.0 using the neighbor-joining method with a bootstrap of 1,000 replicates, using *Caenorhabditis elegans* (CeVg, NP_509305.1) as an outgroup. **(B)** Typical domains of SfVg and Vgs in other insects. The blue triangle stands for amino acid sequence of SfVg.

The sequence of *SfVg* was identified from transcriptome data. The open reading frame (ORF) of *SfVg* (GenBank Accession No. MN229743) encoded a 2,037 amino acid sequence with a theoretical isoelectric point (pI) of 8.43, a predicted molecular weight (Mw) of 226.6 kDa and a signal peptide (MKGITLIFCVIAVAGVSA) at the N-terminus. The amino acid sequence of SfVg contained five copies of consensus RXXR cleavage sites, eight KXXK regions and twenty-five N-glycosylation sites. The conserved motif GLCG was at amino acid 1,853–1,856 (Supplementary File 2). According to the results of BLAST searches of NCBI database, SfVg shares 89.6 and 84.8% similarity with NlVg and LsVg (*Laodelphax striatellus*). A phylogenetic tree of the Vgs of 32 other species places SfVg on the same branch as Vgs from other hemipteran insects, indicating that these share high sequence identity (Figure 1A).

Based on the results of SMART website, the amino acid sequence of SfVg includes three conserved domains; the lipoprotein vitellogenin_N domain (amino acids 24–921), the DUF1943 domain (amino acids 954–1 262) and the VWFD near the C-terminal end of the protein (amino acids 1,678–1,871) (Figure 1B).

### Sequence and Structure of SfVgR in *S. furcifer*a

**FIGURE 2:**
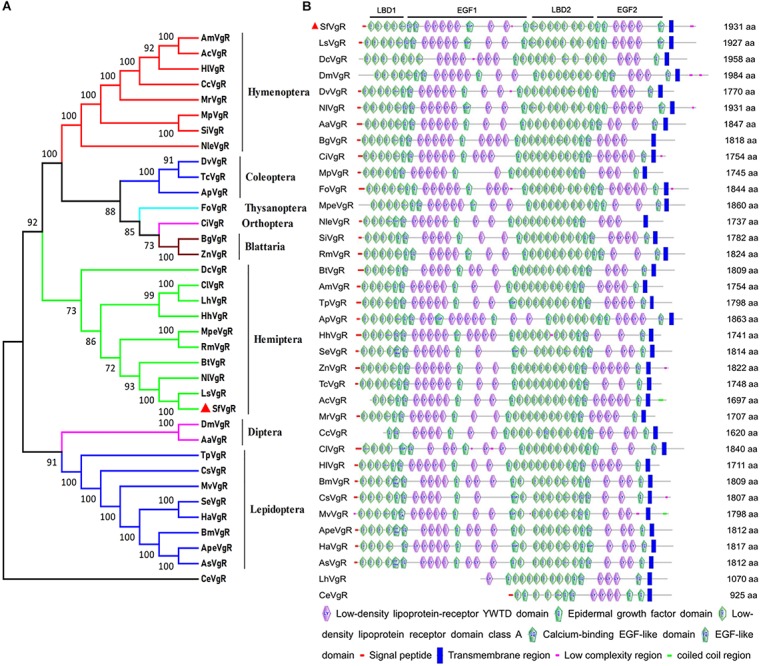
Phylogenetic tree and schematic of primary protein structures of *S. furcifera* vitellogenin receptor (VgR) and VgRs in other insects. **(A)** Phylogenetic tree constructed with MEGA 5.0 using the neighbor-joining method with a bootstrap of 1,000 replicates, using *Caenorhabditis elegans* (CeVgR, AAD56241.1) as an outgroup. **(B)** Typical domains of SfVgR and VgRs in other insects. The red triangle stands for amino acid sequence of SfVgR.

The sequence of the *SfVgR* gene (GenBank Accession No. MN327568) was obtained from transcriptome data and a published *S. furcifera* genome sequence (Wang et al., 2017). The ORF is 5 796 bp and encodes a protein comprised of a 1,931 amino acid sequence. A 19 amino acid signal peptide (MKAIWFLANIVILAAVGFS) was identified in the N-terminal of the putative protein sequence. The theoretical Mw was 215.97 kDa, and the predicted pI was 5.00. A neighbor-joining phylogenetic tree indicates that SfVgR is most closely related to LsVgR (*L. striatellus*) and NlVgR, sharing 82.87 and 77.05% similarity with LsVgR and NlVgR, respectively (Figure 2A).

Analysis of conserved domains indicates that SfVgR is a LDLR superfamily receptor, containing two ligand-binding repeats (LBDs) with five class A (LDLR_A_) repeats in the first LBD (LBD1) and eight repeats in the second LBD (LBD2). Each LBD was followed by an epidermal growth factor (EGF) domain (Figure 2B). Another conserved cluster of acidic residues (CDxxxDCxDGSDE) motif was also found in SfVgR at amino acid 1,089–1,101 (Supplementary File 3). A TM domain spanning amino acids 1,777–1,799, and a cytoplasmic domain spanning amino acids 1,800–1,931, were predicted in the C-terminal end of the protein (Supplementary File 3).

### Differential Expression of SfVg and SfVgR in Different Developmental Stages and Tissues

**FIGURE 3:**
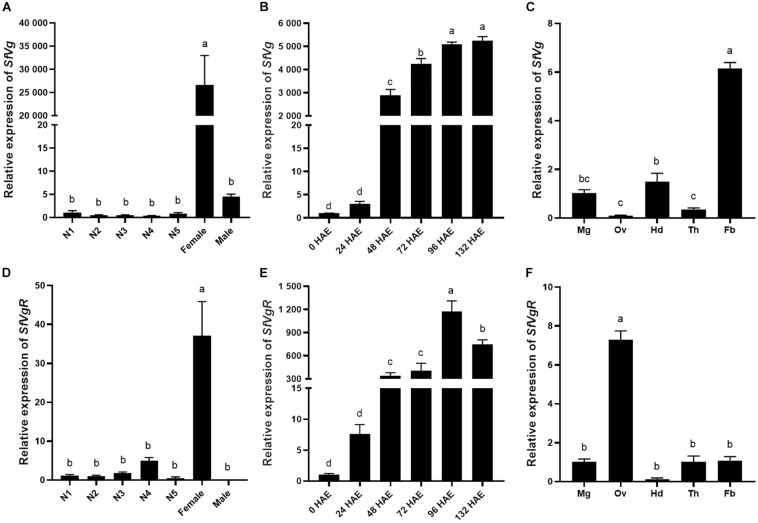
Developmental and tissue-specific expression patterns of *SfVg* and *SfVgR*. Relative expression levels of *SfVg* **(A)** and *SfVgR* **(D)** in the first to fifth nymphal instars (N1, N2, N3, N4, N5), and adults, females and males; Relative expression levels of *SfVg* **(B)** and *SfVgR* **(E)** in the females at different time after emergence; The transcript levels of *SfVg* **(C)** and *SfVgR* **(F)** in various tissues of females. HAE, hours after emergence; Mg, midgut; Ov, ovary; Hd, head; Th, thorax; Fb, fat body. The bar means the average (±SE) of three biological repetitions. *S. furcifera* α*-1 tubulin* (*SfTub*) and *elongation factor 1*α (*SfEF1*α) were used as internal control. Different letters above the bars represent significant differences (ANOVA followed by Tukey’s test, *P* < 0.05).

The transcript levels of *SfVg* and *SfVgR* in different developmental stages and tissues were analyzed using qRT-PCR. *SfVg* was mainly expressed in adult females, there was only trace levels of *SfVg* expression in nymphs and adult males (Figure 3A). The expression of *SfVg* was rapidly increasing 48 h after emergence in adult females (Figure 3B). In 96 h old females, *SfVg* was most highly expressed in the fat body, followed by the head and midgut (Figure 3C).

Expression of *SfVgR* was special highly expressed in adult females. *SfVgR* was found to have a low expression in the nymph insects, but it did not expressed in adult males (Figure 3D). *SfVgR* had an expression profile similar to that of *SfVg* in adult females, in which there was only low expression in the first 24 h after female emergence, and increased sharply to peak 96 h after emergence, then decreased (Figure 3E). In addition, *SfVgR* was mainly expressed in the ovary (Figure 3F).

### Effect of RNAi Knockdown of SfVg and SfVgR

**FIGURE 4:**
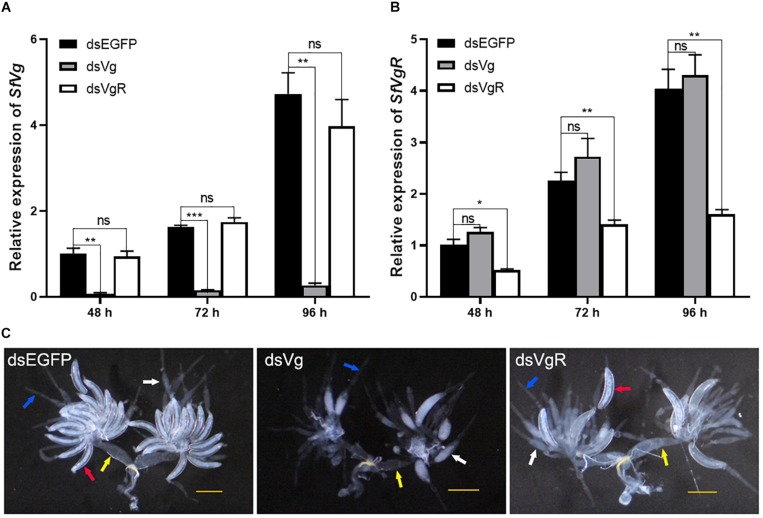
Functional analysis of SfVg and SfVgR in the ovarian development of *S. furcifera* females. Expression levels of *SfVg* **(A)** and *SfVgR* **(B)** in females after injected with dsVg, dsVgR and dsEGFP for 48, 72, and 96 h. Asterisk indicate statistically significant difference (*t*-test: ^∗^*P* < 0.05, ^∗∗^*P* < 0.01, ^∗∗∗^*P* < 0.001). “ns” indicates no significant difference. **(C)** Effect of *SfVg* (dsVg) and *SfVgR* (dsVgR) RNAi on ovarian development with dsEGFP as a control. Ovaries were dissected at 132 h post-injection. *SfEF1a* and *SfTub* were used as internal control. The red, white, blue and yellow arrowheads marked the complete oocytes, incomplete oocytes, ovarioles and oviducts, respectively. Scale bar, 0.5 mm.

A total of 100 ng dsRNAs of either *SfVg*, *SfVgR* and *EGFP* were injected into newly emerged female *S. furcifera*. Expression of *SfVg* in the dsVg treatment group 48, 72, and 96 h after injection was 93.7, 90.5, and 94.3% lower, respectively, than in the dsEGFP control group (Figure 4A). Expression of *SfVgR* in the dsVgR treatment group 48, 72, and 96 h after injection was 48.5, 37.7, and 60.3% lower, respectively, than in the dsEGFP control group (Figure 4B). Injection of dsVg had no significant effect on the expression of *SfVgR* (Figure 4A) and vice versa (Figure 4B).

Microscopic examination of the ovaries of the dsRNA treated females 132 h after injection showed that knockdown of *SfVg* and *SfVgR* caused ovaries to have less Vn in the basal oocyte, there was no complete oocytes in dsVg treated females, and the ovaries of females injected with dsVgR had fewer complete oocytes than that in females injected with dsEGFP (Figure 4C).

## Discussion

Vitellogenesis, including the secretion of Vg in the fat body and its sequestering by maturing oocytes though VgR mediated endocytosis, plays a pivotal role in insect reproduction (Sappington and Raikhel, 1998; Roy et al., 2018). Better understanding of the mechanisms regulating reproduction in insects can potentially identify genes that could be targeted to control insect pests. *S. furcifera* is a notorious pest that damages rice plants by sucking phloem sap and transmitting a virus. However, little is known about the role of Vg and VgR in this species.

We cloned and identified the ORF sequences of *SfVg* and *SfVgR* from *S. furcifera* and analyzed the molecular characteristics of these genes. As expected, the amino acid sequence of SfVg contains the LPD_N, VWD and DUF1943 domains, all of which are highly conserved in most insects (Tufail et al., 2010; Wu et al., 2018; Yao et al., 2018). The motifs GL/ICG, DGXR and K/RXXR/K are also regarded as conserved domains in insect Vg proteins (Sappington and Raikhel, 1998; Upadhyay et al., 2016) and we found a GLCG motif at the C-terminal of SfVg. However, SfVg has no DGXR motif, which is usually located 17–19 residues upstream of the GL/ICG motif in most insect Vg sequences (Tufail and Takeda, 2008; Lee et al., 2015). There were thirteen K/RXXR/K motifs in SfVg which play a vital role in maturation of primary SfVg protein. Most insect primary *Vg* gene products are known to be cleaved in the fat body to produce several subunits and the motif K/RXXR/K, specifically recognized by subtilisin-like endoproteases, acts as the consensus cleavage site (Sappington and Raikhel, 1998; Tufail and Takeda, 2008). In addition, 25 putative glycosylation sites NXS/T and 286 putative phosphorylated residues (*S* = 194, *T* = 55, *Y* = 37) were found in the SfVg amino acid sequence (Supplementary File 4), which indicates that SfVg may be highly phosphorylated (Tufail et al., 2010).

SfVgR is highly homologous with other hemipteran *VgR* genes. Like *NlVgR* (Lu et al., 2015) and *LsVgR* (Figure 2B), *SfVgR* has five LDLR_A_ repeats in the first ligand-binding site and eight in the second. After the second EGF-like domain there is a putative O-linked sugar domain with five threonine and three serine residues at amino acid positions 1,749–1,776. It has been suggested that this serine and threonine enriched region contributes to VgR’s stability and regulates the signal pathway (Willnow, 1999; Tufail and Takeda, 2009). Furthermore, SfVgR is also a highly phosphorylated protein (Tufail et al., 2010) with nine putative glycosylation sites NXS/T (Supplementary File 3) and 186 putative phosphorylated residues (*S* = 101, *T* = 54, *Y* = 31) (Supplementary File 5).

Consistent with their function, both *SfVg* and *SfVgR* were much more highly expressed in adult females. Higher expression of *Vg* and *VgR* in adult females been reported in other insects (Zhong et al., 2015; Zhang W. et al., 2016). However, Vg can no longer be considered a female-specific protein because it has been found to be expressed and synthesized in some male insects, such as *A. mellifera* (Piulachs et al., 2003). We detected trace levels of *SfVg* expression in adult male *S. furcifera* (Figure 3A) but we could not detect any expression of *SfVgR* in males. We did detect some expression of *SfVgR* in nymphs. In fact, previous studies have reported that *VgR* expression begins in later nymph instars in several insects (Shu et al., 2011; Shang et al., 2018).

It has been demonstrated that VgR acts as a receptor mediating the uptake of Vg into maturing oocytes (Roy et al., 2018; Liu et al., 2019). Whereas *SfVgR* was mainly expressed in the ovaries, *SfVg* was highly expressed in fat body. Similar findings have been reported in other insect species. For example, expression of *Vg* is significantly higher in the female fat body of *N. lugens*, *A. citricidus*, and *Spodoptera exigua* than in other tissues (Lu et al., 2015; Zhao et al., 2016; Shang et al., 2018) and *VgR* is highly expressed in the ovary of *N. lugens* (Lu et al., 2015), *B. dorsali* (Cong et al., 2015), and *Helicoverpa armigera* (Zhang W. et al., 2016). Furthermore, previous research has demonstrated the dependence of ovarian development on *VgR* expression in insects (Tufail and Takeda, 2009; Wu et al., 2018), and that *Vg* and *VgR* have similar expression patterns (Lu et al., 2015; Wu et al., 2018). We found that transcription of *SfVg* and *SfVgR* began to increase at 48 h after female emergence and increased sharply 96 h after emergence. The mRNA expression levels of *SfVg* and *SfVgR* are closely correlated with oocyte development of adult females, 96 h after adult females emergence is the key time that Vn largely deposits in oocytes.

A number of previous studies have shown that RNAi is an ideal tool for determining the role of different genes in *S. furcifera* (Jia et al., 2013; Yang et al., 2014; Hu et al., 2019b). We successfully knocked down the expression of *SfVg* and *SfVgR* by injecting dsRNA of these two genes into the thoracic cavity of newly emerged *S. furcifera* females. Depletion of *SfVg* or *SfVgR* caused less yolk protein deposition in oocytes and arrested oocyte maturation. In the bedbug *Cimex lectularius*, RNAi-mediated silencing of *ClVg* also reduced egg production and caused the ovaries to atrophy (Moriyama et al., 2016). Similarly, treating female *H. armigera* with *HaVgR* dsRNA inhibited yolk protein deposition in the ovaries (Zhang W. et al., 2016).

Silencing either *SfVg* or *SfVgR* did not influence the expression of the other (Figures 4A,B). These results are supported by a study on *N. lugens* which found that silencing *NlVg* had no effect on the mRNA transcript and protein levels of *NlVgR*, and that injection of *NlVgR* dsRNA also had no effect on *NlVg* (Lu et al., 2015). This indicates that the low level of NlVg in ovaries was due to the lack of NlVgR to mediate the uptake of NlVg into oocytes, rather than a reduction in NlVg synthesis. Conversely, however, in *A. citricidus* knockdown of *AcVg* decreased the expression of *AcVgR*, whereas silencing *AcVgR* up-regulated the transcript level of *AcVg* (Shang et al., 2018).

This paper provides the ORF sequences of *SfVg* and *SfVgR* in *S. furcifera* and analysis of the expression profiles of these two genes. Furthermore, the results of our RNAi experiments demonstrate that SfVg and SfVgR play a crucial role in oocyte maturation in *S. furcifera*. These findings highlight the potential of targeting *SfVg* and *SfVgR* as a means of controlling *S. furcifera*. For example, plant-mediated RNAi target to these two genes may provide a new strategy to control this pest. In fact, Transgenic cotton plants producing dsRNA that targets the key gene *FATTY ACYL-COA REDUCTASE* (*AsFAR*) in insect reproduction have been designed to control plant bugs (*Adelphocoris suturalis*) (Luo et al., 2017).

## Data Availability Statement

The datasets analyzed in this article are not publicly available. Requests to access the datasets should be directed to KH, wjhk050925@163.com.

## Author Contributions

KH and PT did most of the experimental work. ZL, LY, and HH collected the insects. LQ, YT, and KH participated in the manuscript writing. YL designed the study. KH and WD analyzed the data and wrote the manuscript.

## Conflict of Interest

The authors declare that the research was conducted in the absence of any commercial or financial relationships that could be construed as a potential conflict of interest.

## Footnotes

1 https://blast.ncbi.nlm.nih.gov/

2 http://smart.embl-heidelberg.de/

3 https://web.expasy.org/compute_pi/

4 http://www.cbs.dtu.dk/services/NetNGlyc/

5 http://www.cbs.dtu.dk/services/NetPhos/

6 http://www.cbs.dtu.dk/services/TMHMM/

7 http://www.ncbi.nlm.nih.gov/tools/primer-blast
