# Supplementary material for: Molecular Characterization of Vitellogenin and Its Receptor in Sogatella furcifera, and Their Function in Oocyte Maturation
Source: Front Physiol. 2019 Dec 19;10:1532. doi: 10.3389/fphys.2019.01532 (PMC6930921; doi:10.3389/fphys.2019.01532)
Supplement: Supplementary file 1 [file Table_1.DOCX]

**Supplementary file 1. The list of the other insect species that were used for sequence comparisons and phylogenetic analysis of SfVg and SfVgR.**

CeVg, *Caenorhabditis elegans*, NP_509305.1;

HhVg, *Halyomorpha halys*, XP_014270535.1;

GpVg, *Geocoris pallidipennis*, ALN70475.1;

TcaVg, *Trigonotylus caelestialium*, BAJ33507.1;

AlVg, *Apolygus lucorum*, AGT39945.1;

NlVg, *Nilaparvata lugens*, AEL22916.1;

SfVg, *S. furcifera*, MN229743;

LsVg, *Laodelphax striatellus*, AGJ26478.1;

BtVg, *Bemisia tabaci*, ADU04393.1;

CseVg, *Chrysopa septempunctata*, AGJ71349.1;

PaVg, *Periplaneta americana*, BAA86656.1;

TmVg, *Tenebrio molitor*, AAU20328.2;

TcVg, *Tribolium castaneum*, XP_971398.1;

AaVg, *Aedes aegypti*, AAA18221.1;

CqVg, *Culex quinquefasciatus*, AAV31930.1;

AgVg, *Anopheles gambiae*, AAF82131.1;

EfVg, *Encarsia Formosa*, AAT48601.1;

PvVg, *Polyrhachis vicina*, AUG84084.1;

SiVg, *Solenopsis invicta*, AAP47155.1;

BlVg, *Bombus lantschouensis*, AUX13057.1;

BhVg, *Bombus hypocrite*, ACU00433.1;

BiVg, *Bombus ignites*, ACQ91623.1;

OcVg, *Osmia cornifrons*, AIU68826.1;

AcVg, *Apis cerana*, AYN79154.1;

AmVg, *Apis mellifera*, NP_001011578.1;

LdVg, *Lymantria dispar*, AAC02818.1;

MvVg, *Maruca vitrata*, AXY55008.1;

CsVg, *Chilo suppressalis*, AMD78107.1;

BmVg, *Bombyx mori*, NP_001037309.1;

HaVg, *Helicoverpa armigera*, AGL08685.1;

SeVg, *Spodoptera exigua*, AOH73254.1;

SlVg, *Spodoptera litura*, ABU68426.1;

BgVg, *Blattella germanica*, CAA06379.2.

CeVgR, *Caenorhabditis elegans*, AAD56241.1;

AmVgR, *A. mellifera*, XP_026295652.1;

AcVgR, *Apis cerana cerana*, PBC31775.1;

HlVgR, *Habropoda laboriosa*, KOC62359.1;

MrVgR, *Megachile rotundata*, XP_012143749.1;

CcVgR, *Ceratina calcarata*, XP_026667852.1;

NleVgR, *Neodiprion lecontei*, XP_015509591.1;

MpVgR, *Monomorium pharaonis*, XP_012532761.1;

SiVgR, *S. invicta*, AAP92450.1;

BgVgR, *B. germanica*, CAJ19121.1;

ZnVgR, *Z. nevadensis*, XP_021934248.1;

CiVgR, *Calliptamus italicus*, QBM78333.1;

FoVgR, *Frankliniella occidentalis*, XP_026271484.1;

LsVgR, *L. striatellus*, QBF03701.1;

NlVgR, *N. Lugens*, XP_022195000.1;

BtVgR, *B. tabaci*, ADM34986.1;

MpeVgR, *Myzus persicae*, XP_022162173.1;

RmVgR, *Rhopalosiphum maidis*, XP_026805520.1;

HhVgR, *H. halys*, XP_014291555.1;

ClVgR, *C. lectularius*, XP_014250447.1;

LhVgR, *Lygus hesperus*, JAF98697.1;

DcVgR, *Diaphorina citri*, XP_026689064.1;

DmVgR, *Drosophila melanogaster*, AAB60217.1;

AaVgR, *A. aegypti*, AAK15810.1;

ApVgR, *Agrilus planipennis*, XP_025835220.1;

TcVgR, *T. castaneum*, XP_015837722.1;

DvVgR, *Diabrotica virgifera*, AQS83398.1;

TpVgR, *T. pui*, AWJ95281.1;

CsVgR, *Conopomorpha sinensis*, ASX95053.1;

MvVgR, *M. vitrata*, AXY55007.1;

SeVgR, *S. exigua*, AOX13593.1;

HaVgR, *H. armigera*, AGF33811.2;

ApeVgR, *Antheraea pernyi*, AEJ88360.1;

BmVgR, *B. mori*, ADK94452.1;

AsVgR, *Actias selene*, AFV32171.1.
