# Supplementary material for: Molecular Characterization of Vitellogenin and Its Receptor in Sogatella furcifera, and Their Function in Oocyte Maturation
Source: Front Physiol. 2019 Dec 19;10:1532. doi: 10.3389/fphys.2019.01532 (PMC6930921; doi:10.3389/fphys.2019.01532)
Supplement: Supplementary file 2 [file Table_2.DOCX]

**Supplementary file 2.** **Protein sequence of *Sogatella furcifera* vitellogenin.** N-terminus signal peptide, consensus subtilisin-like endoproteases cleavage sites R/KXXR/K and GL/ICG motif are highlighted by yellow, green and gray colours, respectively. Asn-Xaa-Ser/Thr sequons (NXT/S) in the sequence output below are highlighted in blue. Asparagines predicted to be N-glycosylated are highlighted in red.

**MKGITLIFCVIAVAGVSASGNGPWNSNQLYHYRVQGRTLSAMHQAGSPQMVGIHIKADLTVQAKNENQAVFKISNAQYAD 80**

**VHQNLTGGWQQELRSNELQYKQLPLSQANHAFEVNYKQGSVRSLQVNKNTPTWELNMIKGFVSLFQVDVTGQNVIKSRRN 160**

**IVPNSNQVSGSFKAMEDSVTGKCETHYDVDLLPMRVVQEHPEIAPFAVHQQQQQQHNLIQVVKSRNFSNCDNPVTYHFGF 240**

**TQETNWEPASNQMGNLVNRASTSRIILSGQPNSFTIQSSVTQNEIAISPFGYNQQKGVVGTLMNATLVSMSHASGSPQSV 320**

**QNAQKINDLVYEFNPASNSDNNGANNRRSSNYNRQNDNDSSSSSSSSSSSSSSSDSSSSSSSSEENYNKNGKNNNNKSGK 400**

**NNNWNKNNNNNKWNNDDDDTNRYNSNNNRHQNNDNDDDAYWRSQQKTKSRSRRSILKNFNNDNDDDVDNQNNRNYNNQNR 480**

**NNENDSSEENNDNNQNNKNRNNRQNDNNNKYYNNKNNKNRNNNDNDDSSSSSSSDSSSSSSSSSSSSSSSSSSSSSSSDL 560**

**DSSEENWQQKPDMNDAPSTPFLPHFVGVRGNSIQADKQIDIVNEAQKVAMRIGAQVQKPSAIPGQNTLTSFTILTRMIQT 640**

**MSAKQIQEVKQRLFIDRNNANGKSSADAKKLQSWEAFKHATANAGTGPALEAIKNWVEKGDVRNEKAAELVAVLPRTARL 720**

**PTDKYIKTFFQFATSSNVVNQKYLNSTIIIGFSEILRKAQVDSDNKHMRYGVHSFGHLTSKNDQSLQQEYLPYLEEKLKS 800**

**AFEKGDSQKIIVYIQALGNTAHPRLLKTFEPYLEGKKSASRFQRLLMVASLYQMTRVHPTTARAVLYRIYKNPGEAPELR 880**

**VAALHLLANTNPPAAMLQRIAQQTNWEQSKQVISATQSFIRSAANMDQNPDSVEFARNAQSAVDMLNPADYGYSMSKNYL 960**

**SSYVIDNIDKSYESQISSIGSFDSIFPSSVFVNFMANDGGYKHQVFHHSAMFSSVNDLLELVNTQFKNNNNNNSNHRNNN 1040**

**RSGSHDNEDNHSNRNSNNEWTAENVFKALNIQKDQAEQLEGNIFLTMLGGKRAFAINNHTIEKIPAIFKEAAQKLKHTSF 1120**

**NLTQFYSKNTMKVAFPTPMGLPFVYASSVPTMVYVGGETKVNSHPDLANGNNNFVNIPQYVNMSADIEAVYSMQANSKFG 1200**

**TVAPFNHHEYYASVERNIQFYTAVQMDANIDIDNKAVELRVQPLNKEDKQNVFQYSTVLFTTKSNILNFNPALQEEGTER 1280**

**VHVGKAKQIQQNFGKDSTGFAFEASYWSEKGFGDLASLYEEVSKFDVQSALTSPWVQSSLNPNNITVAFKPSQSSSKVAK 1360**

**FTFSYSDNSNSNNNSNNHNDNNSHDSNNNRADSSAAHPSSTAANSVSRQNEFLHKVASGISGANAMVVDVSAKFQDNHGQ 1440**

**SSAQYVATLALANSDASPNARVLFFASMDPANSGSSSLSKAQVCAAAASHFPNVPLMNFNDALKANPDSHITAEVAFGSN 1520**

**CNAGGHIRADAKLSQTQEFQDFAKNRPMAKKCFQLIQKGQALEYACQNATKVANMLNKYDVSIKYDRVPNAFKNVTYNVY 1600**

**SALAQVAFPYHSENMFSQHSNPSGKIDLNARFNYNLRYFNASINSPFFTANFKNVEVDPAVRPLVIFHPSLNSLELMSYN 1680**

**ENYDYPTCSVSKNSISTFDNKTYSADLEGWHVMFASTPKNYNDNSGRYSVSNSQSNSFYKYKKVAILAKNAGSQRKAVKM 1760**

**LLGDNVIDINPSGSDSNNNSPNVNVQVNGNKMNIANNRLASFDDFDGETLVEISVTDNGEVQVQSPSHGIAVNHDGANFM 1840**

**IDADSYYSGEVRGLCGTYSGDKYTDFTTPKKCILREAKLFAATYALSGSSSNVEQLKRQAEQVTCFKRHPIFADVITSND 1920**

**YDRSNNNNNSNNRNRNSKISSMKYQVSDSSSSIELVQDIKNIDDQVCFSIRPIPRCQQGSSPVGSSEKEVQYLCISHGKN 2000**

**AAYWSGEIRRGAYVNFEQKQPNATFKKNIPQRCVREN**
