# Supplementary material for: Molecular Characterization of Vitellogenin and Its Receptor in Sogatella furcifera, and Their Function in Oocyte Maturation
Source: Front Physiol. 2019 Dec 19;10:1532. doi: 10.3389/fphys.2019.01532 (PMC6930921; doi:10.3389/fphys.2019.01532)
Supplement: Supplementary file 3 [file Table_3.DOCX]

**Supplementary file 3.** **Protein sequence of vitellogenin.** N-terminus signal peptide is highlighted by gray colour. Asn-Xaa-Ser/Thr sequons (NXT/S) in the sequence output below are highlighted in blue. Asparagines predicted to be N-glycosylated are highlighted in red. Transmembrane region is underlined. Cytoplasmic region is highlighted in deep red. O-Linked sugar domain is highlighted in orange. The conserved cluster of acidic residues motif (CDxxxDCxDGSDE) is highlighted in yellow.

**MKAIWFLANIVILAAVGFSTENFEGTLPDGGCPLDHFTCDDGECILLEQHCDHQDDCSDRSDEKNCDLNFCLEPDYFKCG 80**

**NGNCIDAGMVCNSVRDCRDGSDEDPERCHSDIDYPDHWYENKKCDLLQFTCEDKMCIPLEWVCDGEANCLDESDEKASLC 160**

**AKHHCFPGQFRCQDGLCIEEDFKCDGTADCHDGSDEENCPVPVYSVDEECNLDNEKYLCKDRQLCIDISKLCNGHRDCFD 240**

**GSDEDGKCHEKVECNDANCTIEQCFPSPSGQFCLCNKGYKHEKGVCIDINECEEFGICDQKCNNTVGGYRCSCDQGYLLQ 320**

**KDGHSCRGEGNYEPMIYFSLGNEIRVRFLKSGMYHSLASNLTQAIGVEVHKHHVYWTNIFEGQESIVRAQQLGAERTPIV 400**

**TAGLSEPEDLAVDWITGNIYFTDSGNKFIGVCSEDVKHCTVLHNRDINNPRGIALLPYEGYMYWSDWGDKSVIARSGMDG 480**

**LDVVDFVSADLGWPNGITIDHGNQRLYWVDAKLASIESVRLDGKDRRKVLEHALAHPYSIAVFEDTIYWSDWQTLKIESA 560**

**NKFTGKNRRTVVQDKKKIFGIKVYHPAMYDLNEINYCFAAPCSDLCLLAPSREDPLGPPGPPARRYTCACPEGKELSANN 640**

**YSCIATDKEQVMVIGNRDRLFHYEHKQLGKVHVDEIPLDQNTAMIGDIGALQYNSLDGSLIVGDSYNRKMTSVDLKTLQT 720**

**RDIVTSGVGRIEGIAFDNLGANIYWTDSELGKVEVINTINHHRKTILNHLQGDIPRGIAVIPSEGVMYVSLNSPISAHID 800**

**KLSMSGDVMSRTHIFEENLRGPFLPLFYDQMTHRMFWADSGMRRIEHTSWNGEERHTYKELSSSPISISSVGVDIFWSTA 880**

**GYKSISYASKLLNKETRILDLTNHVYSNSKIFLTSVTGIDSRRNHPCAFSNGNCSHICLVKSEKLGECACPDGMFLKEGG 960**

**MQCEVKAACEVTEFECEVSSVAGVARHCIPLKKKCDGHRDCPMGEDEDRAICMAKDFIKLQCLPNQFACLDGQKCIAEAQ 1040**

**VCNWIADCDDHSDETSGCEHSRMEPNSTECEFRCGGASSTECVLESQRCDMQPDCSDGSDELNCDKHLCDTQSQFRCKSG 1120**

**NCISKEMECNGEMDCRDGSDEHNKCNHVRTCSPLQITCDNGQCIDKELKCNGRNDCDDASDEHSCPQRSVPHIPIFQHPG 1200**

**ASSQQAVECDTRFEFECERGHCIPSTARCNHTSECRNGEDELNCMGCHRDQFQCKNERCIYHTWVCDGKNDCGDNSDEEV 1280**

**ALCKARSIDKSDAHSVAASNDLHCFGSFRCTSDVNECVHTDKVCNGEKDCSDGSDEGGMCFKGCENAGCSDGCQKTPHGP 1360**

**KCTCPKGFELTGDAKTCADIDECATEQYCSQYCSNTPGAFRCSCKAPEYVLRENGMSCRAKGGEMQFVYSVYNEIRTMTG 1440**

**WHSYLGIIHTDPDYRARVEGLTADVRRKQVYWTTATNDSLYAISMDNRRLIRSARIQRPSRLSIDWITGNVYVVEASSQI 1520**

**TAVNFDKRSYARLYKSDPAKDIEALAVDPVMRTMFWSEKLAHKIQKSTIFRADTSGSNVVELVTADLKQVSDIFIDSFHN 1600**

**QIYWADSITKKVERAAFDGSNRREVFTSPDVPTDITIFEDYIYVMVQADSPTEVKEMEDTGNVWRCGLYGAAFEKCELFR 1680**

**IHPKHFTVPYHFDIMHPGLQLRGHNDCLNATDCENAGGMCLLRNHKLRPSAVCVCADGTRMRKNSVCATTTEQDDIFSGA 1760**

**DFLTSNQHNLQTGFGSVWWMLLGFVFVVLPVVGILLFVYRGGPSAPINPPQWMPGFCTRRFPFHTIRFNSKFGNIDADDT 1840**

**IPAYSDFQFHPCQLNPGEHQYENPIAAMQAEQNGAISIKTMNEIDIQMGEEKNGWLGGEGGAPHGSGEDSDSSSIIEMSK 1920**

**VNGEESRTLLL**
