# Supplementary material for: Molecular Characterization of Vitellogenin and Its Receptor in Sogatella furcifera, and Their Function in Oocyte Maturation
Source: Front Physiol. 2019 Dec 19;10:1532. doi: 10.3389/fphys.2019.01532 (PMC6930921; doi:10.3389/fphys.2019.01532)
Supplement: Supplementary file 4 [file Table_4.DOCX]

**Supporting file 4. The putative phosphorylated residues in SfVg.**

MKGITLIFCVIAVAGVSASGNGPWNSNQLYHYRVQGRTLSAMHQAGSPQM # 50

VGIHIKADLTVQAKNENQAVFKISNAQYADVHQNLTGGWQQELRSNELQY # 100

KQLPLSQANHAFEVNYKQGSVRSLQVNKNTPTWELNMIKGFVSLFQVDVT # 150

GQNVIKSRRNIVPNSNQVSGSFKAMEDSVTGKCETHYDVDLLPMRVVQEH # 200

PEIAPFAVHQQQQQQHNLIQVVKSRNFSNCDNPVTYHFGFTQETNWEPAS # 250

NQMGNLVNRASTSRIILSGQPNSFTIQSSVTQNEIAISPFGYNQQKGVVG # 300

TLMNATLVSMSHASGSPQSVQNAQKINDLVYEFNPASNSDNNGANNRRSS # 350

NYNRQNDNDSSSSSSSSSSSSSSSDSSSSSSSSEENYNKNGKNNNNKSGK # 400

NNNWNKNNNNNKWNNDDDDTNRYNSNNNRHQNNDNDDDAYWRSQQKTKSR # 450

SRRSILKNFNNDNDDDVDNQNNRNYNNQNRNNENDSSEENNDNNQNNKNR # 500

NNRQNDNNNKYYNNKNNKNRNNNDNDDSSSSSSSDSSSSSSSSSSSSSSS # 550

SSSSSSSSDLDSSEENWQQKPDMNDAPSTPFLPHFVGVRGNSIQADKQID # 600

IVNEAQKVAMRIGAQVQKPSAIPGQNTLTSFTILTRMIQTMSAKQIQEVK # 650

QRLFIDRNNANGKSSADAKKLQSWEAFKHATANAGTGPALEAIKNWVEKG # 700

DVRNEKAAELVAVLPRTARLPTDKYIKTFFQFATSSNVVNQKYLNSTIII # 750

GFSEILRKAQVDSDNKHMRYGVHSFGHLTSKNDQSLQQEYLPYLEEKLKS # 800

AFEKGDSQKIIVYIQALGNTAHPRLLKTFEPYLEGKKSASRFQRLLMVAS # 850

LYQMTRVHPTTARAVLYRIYKNPGEAPELRVAALHLLANTNPPAAMLQRI # 900

AQQTNWEQSKQVISATQSFIRSAANMDQNPDSVEFARNAQSAVDMLNPAD # 950

YGYSMSKNYLSSYVIDNIDKSYESQISSIGSFDSIFPSSVFVNFMANDGG # 1000

YKHQVFHHSAMFSSVNDLLELVNTQFKNNNNNNSNHRNNNRSGSHDNEDN # 1050

HSNRNSNNEWTAENVFKALNIQKDQAEQLEGNIFLTMLGGKRAFAINNHT # 1100

IEKIPAIFKEAAQKLKHTSFNLTQFYSKNTMKVAFPTPMGLPFVYASSVP # 1150

TMVYVGGETKVNSHPDLANGNNNFVNIPQYVNMSADIEAVYSMQANSKFG # 1200

TVAPFNHHEYYASVERNIQFYTAVQMDANIDIDNKAVELRVQPLNKEDKQ # 1250

NVFQYSTVLFTTKSNILNFNPALQEEGTERVHVGKAKQIQQNFGKDSTGF # 1300

AFEASYWSEKGFGDLASLYEEVSKFDVQSALTSPWVQSSLNPNNITVAFK # 1350

PSQSSSKVAKFTFSYSDNSNSNNNSNNHNDNNSHDSNNNRADSSAAHPSS # 1400

TAANSVSRQNEFLHKVASGISGANAMVVDVSAKFQDNHGQSSAQYVATLA # 1450

LANSDASPNARVLFFASMDPANSGSSSLSKAQVCAAAASHFPNVPLMNFN # 1500

DALKANPDSHITAEVAFGSNCNAGGHIRADAKLSQTQEFQDFAKNRPMAK # 1550

KCFQLIQKGQALEYACQNATKVANMLNKYDVSIKYDRVPNAFKNVTYNVY # 1600

SALAQVAFPYHSENMFSQHSNPSGKIDLNARFNYNLRYFNASINSPFFTA # 1650

NFKNVEVDPAVRPLVIFHPSLNSLELMSYNENYDYPTCSVSKNSISTFDN # 1700

KTYSADLEGWHVMFASTPKNYNDNSGRYSVSNSQSNSFYKYKKVAILAKN # 1750

AGSQRKAVKMLLGDNVIDINPSGSDSNNNSPNVNVQVNGNKMNIANNRLA # 1800

SFDDFDGETLVEISVTDNGEVQVQSPSHGIAVNHDGANFMIDADSYYSGE # 1850

VRGLCGTYSGDKYTDFTTPKKCILREAKLFAATYALSGSSSNVEQLKRQA # 1900

EQVTCFKRHPIFADVITSNDYDRSNNNNNSNNRNRNSKISSMKYQVSDSS # 1950

SSIELVQDIKNIDDQVCFSIRPIPRCQQGSSPVGSSEKEVQYLCISHGKN # 2000

AAYWSGEIRRGAYVNFEQKQPNATFKKNIPQRCVREN # 2050

..................S..................T.S......S... # 50

.........T.............S...Y.......T.............Y # 100

.....S.........Y...S.........T............S....... # 150

......S.............S......S.T....T.Y............. # 200

...........................S............T..T...... # 250

..........ST.....S..........S.T................... # 300

T.......S.S..S.S..............Y.....S.S.........SS # 350

.........SSSSSSSSSSSSSSS.SSSSSSSS...Y..........S.. # 400

...................T..Y.S..............Y..S...T.S. # 450

S..S....................Y..........SS............. # 500

..........YY...............SSSSSSS.SSSSSSSSSSSSSSS # 550

SSSSSSSS...SS..............ST............S........ # 600

...................S......T............T.S........ # 650

.............SS.......S........................... # 700

................T....T..Y..T......SS......Y..S.... # 750

..S................Y...S....TS....S..............S # 800

......S............T.......T.........S.S.........S # 850

.........TT............................T.......... # 900

...T.........S.T.S...S.........S........S......... # 950

...S......S.Y.......SY.S..S...S.......S........... # 1000

...........................................S...... # 1050

.S...S.............................T.............. # 1100

.................T....T...S..T......T............. # 1150

............S................Y................S... # 1200

T.........Y.........Y............................. # 1250

......T...T................T...................T.. # 1300

.....Y.S........S..............TS....S.......T.... # 1350

.S..SS.....T.SY...S.......................S....... # 1400

T...S.........................S.........S...Y..... # 1450

...S..S...............S...S.S..................... # 1500

........S........................S.T.............. # 1550

...................T...........S..Y..........T.... # 1600

................S..S..S..............Y......S..... # 1650

...................S.......S....Y.Y.T.S....S.ST... # 1700

..YS...........ST..........YS...S.S.S.Y.Y......... # 1750

..S..................S.S.S...S.................... # 1800

S.......T....S.T........S.S.................SY.S.. # 1850

......T.S...Y...TT..............T...S...S......... # 1900

.................S..Y........S......S..SS..Y..S.S. # 1950

SS................S...........S...SS.....Y...S.... # 2000

..Y.........Y..........T.............
