# Supplementary material for: Molecular Characterization of Vitellogenin and Its Receptor in Sogatella furcifera, and Their Function in Oocyte Maturation
Source: Front Physiol. 2019 Dec 19;10:1532. doi: 10.3389/fphys.2019.01532 (PMC6930921; doi:10.3389/fphys.2019.01532)
Supplement: Supplementary file 5 [file Table_5.DOCX]

**Supporting file 5. The putative phosphorylated residues in SfVgR.**

MKAIWFLANIVILAAVGFSTENFEGTLPDGGCPLDHFTCDDGECILLEQH # 50

CDHQDDCSDRSDEKNCDLNFCLEPDYFKCGNGNCIDAGMVCNSVRDCRDG # 100

SDEDPERCHSDIDYPDHWYENKKCDLLQFTCEDKMCIPLEWVCDGEANCL # 150

DESDEKASLCAKHHCFPGQFRCQDGLCIEEDFKCDGTADCHDGSDEENCP # 200

VPVYSVDEECNLDNEKYLCKDRQLCIDISKLCNGHRDCFDGSDEDGKCHE # 250

KVECNDANCTIEQCFPSPSGQFCLCNKGYKHEKGVCIDINECEEFGICDQ # 300

KCNNTVGGYRCSCDQGYLLQKDGHSCRGEGNYEPMIYFSLGNEIRVRFLK # 350

SGMYHSLASNLTQAIGVEVHKHHVYWTNIFEGQESIVRAQQLGAERTPIV # 400

TAGLSEPEDLAVDWITGNIYFTDSGNKFIGVCSEDVKHCTVLHNRDINNP # 450

RGIALLPYEGYMYWSDWGDKSVIARSGMDGLDVVDFVSADLGWPNGITID # 500

HGNQRLYWVDAKLASIESVRLDGKDRRKVLEHALAHPYSIAVFEDTIYWS # 550

DWQTLKIESANKFTGKNRRTVVQDKKKIFGIKVYHPAMYDLNEINYCFAA # 600

PCSDLCLLAPSREDPLGPPGPPARRYTCACPEGKELSANNYSCIATDKEQ # 650

VMVIGNRDRLFHYEHKQLGKVHVDEIPLDQNTAMIGDIGALQYNSLDGSL # 700

IVGDSYNRKMTSVDLKTLQTRDIVTSGVGRIEGIAFDNLGANIYWTDSEL # 750

GKVEVINTINHHRKTILNHLQGDIPRGIAVIPSEGVMYVSLNSPISAHID # 800

KLSMSGDVMSRTHIFEENLRGPFLPLFYDQMTHRMFWADSGMRRIEHTSW # 850

NGEERHTYKELSSSPISISSVGVDIFWSTAGYKSISYASKLLNKETRILD # 900

LTNHVYSNSKIFLTSVTGIDSRRNHPCAFSNGNCSHICLVKSEKLGECAC # 950

PDGMFLKEGGMQCEVKAACEVTEFECEVSSVAGVARHCIPLKKKCDGHRD # 1000

CPMGEDEDRAICMAKDFIKLQCLPNQFACLDGQKCIAEAQVCNWIADCDD # 1050

HSDETSGCEHSRMEPNSTECEFRCGGASSTECVLESQRCDMQPDCSDGSD # 1100

ELNCDKHLCDTQSQFRCKSGNCISKEMECNGEMDCRDGSDEHNKCNHVRT # 1150

CSPLQITCDNGQCIDKELKCNGRNDCDDASDEHSCPQRSVPHIPIFQHPG # 1200

ASSQQAVECDTRFEFECERGHCIPSTARCNHTSECRNGEDELNCMGCHRD # 1250

QFQCKNERCIYHTWVCDGKNDCGDNSDEEVALCKARSIDKSDAHSVAASN # 1300

DLHCFGSFRCTSDVNECVHTDKVCNGEKDCSDGSDEGGMCFKGCENAGCS # 1350

DGCQKTPHGPKCTCPKGFELTGDAKTCADIDECATEQYCSQYCSNTPGAF # 1400

RCSCKAPEYVLRENGMSCRAKGGEMQFVYSVYNEIRTMTGWHSYLGIIHT # 1450

DPDYRARVEGLTADVRRKQVYWTTATNDSLYAISMDNRRLIRSARIQRPS # 1500

RLSIDWITGNVYVVEASSQITAVNFDKRSYARLYKSDPAKDIEALAVDPV # 1550

MRTMFWSEKLAHKIQKSTIFRADTSGSNVVELVTADLKQVSDIFIDSFHN # 1600

QIYWADSITKKVERAAFDGSNRREVFTSPDVPTDITIFEDYIYVMVQADS # 1650

PTEVKEMEDTGNVWRCGLYGAAFEKCELFRIHPKHFTVPYHFDIMHPGLQ # 1700

LRGHNDCLNATDCENAGGMCLLRNHKLRPSAVCVCADGTRMRKNSVCATT # 1750

TEQDDIFSGADFLTSNQHNLQTGFGSVWWMLLGFVFVVLPVVGILLFVYR # 1800

GGPSAPINPPQWMPGFCTRRFPFHTIRFNSKFGNIDADDTIPAYSDFQFH # 1850

PCQLNPGEHQYENPIAAMQAEQNGAISIKTMNEIDIQMGEEKNGWLGGEG # 1900

GAPHGSGEDSDSSSIIEMSKVNGEESRTLLL # 1950

..................ST.....T...........T............ # 50

.......S..S..............Y................S....... # 100

S........S...Y....Y............................... # 150

..S........................................S...... # 200

....S...........Y........................S........ # 250

................S................................. # 300

...........S............S......Y......S........... # 350

S..Y.S.....T..............T.......S...........T... # 400

....S..............Y.T..........S................. # 450

............Y.S.....S....S...........S............ # 500

..............S..S...........................T.Y.S # 550

...T.........T.....T.........................Y.... # 600

..........S...............T.............Y....T.... # 650

.................................................. # 700

....S.....TS.......T.....S...................T.... # 750

..............T......................Y....S....... # 800

..S.S......T...................T.......S.......TS. # 850

......TY...SSS..S.S.........T..Y.S.SY.S........... # 900

.T...YS.......S.....S........S...........S........ # 950

.....................T............................ # 1000

.................................................. # 1050

.S..TS....S.....ST.........SST.....S.........S..S. # 1100

..........T.S.....S...................S........... # 1150

.S....T......................S.................... # 1200

..S.......T.............ST......S................. # 1250

..........Y.T............S..........S...S...S..... # 1300

......S...TS..................S..S................ # 1350

.....T..............T....T...........Y.S.Y.S.T.... # 1400

..S.....Y.......S............S.Y......T........... # 1450

...Y................Y.......S.Y..S........S......S # 1500

..S........Y.....S..........SY.................... # 1550

.................T.....TS.S.............S......... # 1600

......S.T..........S......TS....T..T....Y.Y......S # 1650

.T.......T........Y.................T............. # 1700

..........T..................S........T.....S...TT # 1750

T....................T...S........................ # 1800

.................T......T....S.................... # 1850

..........Y....................................... # 1900

.....S...S.S.S....S............
